# Porcine *methionine sulfoxide reductase B3:* molecular cloning, tissue-specific expression profiles, and polymorphisms associated with ear size in *Sus scrofa*

**DOI:** 10.1186/s40104-015-0060-x

**Published:** 2015-12-30

**Authors:** Yuebo Zhang, Jing Liang, Longchao Zhang, Ligang Wang, Xin Liu, Hua Yan, Kebin Zhao, Huibi Shi, Tian Zhang, Na Li, Lei Pu, Lixian Wang

**Affiliations:** Key Laboratory of Farm Animal Genetic Resources and Germplasm Innovation, Ministry of Agriculture, Institute of Animal Science, Chinese Academy of Agricultural Sciences, Beijing, 100193 China; Jilin Academy of Agricultural Sciences, Changchun, 130033 China

**Keywords:** Association study, Ear size, Expression, *MSRB3* cloning, Pig

## Abstract

**Background:**

In *Sus scrofa*, *methionine sulfoxide reductase B3* (*MSRB3*) is a crucial candidate gene for ear size, and an important conformational trait of pig breeds. However, challenges in *MSRB3* cDNA amplification have prevented further identification of *MSRB3* allelic variants influencing pig ear size.

**Results:**

We cloned a full-length cDNA sequence of porcine *MSRB3* by rapid-amplification of cDNA ends. The 3,765-bp gene contained a 5’-untranslated region (UTR) (190 bp), a coding region (552 bp), and a 3’-UTR (3,016 bp) and shared 84 %, 84 %, 87 %, 86 %, and 70 % sequence identities with human, orangutan, mouse, chicken, and zebrafish, respectively. The gene encoded a 183-amino acid protein, which shared 88 %, 91 %, 89 %, 86 %, and 67 % identities with human, orangutan, mouse, chicken, and zebrafish, respectively. Tissue expression analysis using qRT-PCR revealed that *MSRB3* was expressed in the heart, liver, lung, kidney, spleen, ear, muscle, fat, lymph, skeletal, and hypothalamic tissues. Three single nucleotide polymorphisms (SNPs) were identified in *MSRB3*: c.-735C > T in the 5’ flanking region, c.2571 T > C in the 3’-UTR, and a synonymous mutation of c.484 T > C in the coding region. The SNPs c.-735C > T and c.2571 T > C were significantly associated with ear size in a Large White × Minzhu F2 population other than in Beijing Black pigs. Subsequently, at SNP c.-735C > T, the mRNA of *MSRB3* was significantly higher expressed in ears of individuals with the TT genotype (Minzhu) than those with CC (Large White).

**Conclusions:**

The porcine *MSRB3* owned a 3,765-bp full-length cDNA sequence and was detected to express in ear tissue. Two SNPs of this gene were shown to be significantly associated with ear size in a Large White × Minzhu intercross population instead of Beijing Black pig population. What’s more, the individuals with higher mRNA expression of *MSRB3* have larger ear sizes. These results provide useful information for further functional analyses of *MSRB3* influencing ear size in pigs.

**Electronic supplementary material:**

The online version of this article (doi:10.1186/s40104-015-0060-x) contains supplementary material, which is available to authorized users.

## Background

In traditional Chinese culture, large ears are considered to be a blessed characteristic. Historically, the large ear trait has been selected for domestic pigs; consequently, most Chinese pig breeds today have ears larger than their foreign counterparts. Ear size is an important trait to distinguish pig breeds [1], and many studies have investigated its genetic basis. Quantitative trait loci (QTL) for ear size have been mapped to *Sus scrofa* chromosomes (SSC) 1, 4, 5, 6, 7, 8, 9, 11, 12, 16, and X [2, 3]. On SSC7, *PPARD* has been identified as a major effect gene; a G32E mutation in this gene is the causal variant conferring the phenotype [4]. Li et al. refined the QTL (11-cM interval) on SSC5 to an 8.7-cM interval using a Duroc × Erhualian intercross population [5]. In a previous genome-wide association study, *MSRB3* is adjacent to the most significant SNP associated with porcine ear size [6]. Moreover, a GWAS using 12 dog breeds with pricked ears and 15 breeds with dropped ears suggested that *MSRB3* is adjacent to the most strongly associated SNP with ear morphology as well [7]. In another study for dogs, single-marker analysis showed *MSRB3* is also near the strongest association with ear floppiness [8]. Therefore, *MSRB3* should be regarded as a good candidate on SSC5 for porcine ear size. However the lack of a full-length cDNA of porcine *MSRB3* has made it challenging to verify its mechanism in influencing ear size. Hence, our study’s aims were to clone porcine *MSRB3*, investigate its transcript abundance and tissue expression, and identify polymorphisms associated with ear size in different populations.

## Methods

### Ethics statement

All animals were treated according to the guidelines for the experimental animals established by the Council of China. All animal experiments were approved by the Science Research Department of the Institute of Animal Science, Chinese Academy of Agricultural Sciences (CAAS) (Beijing, China).

### Sampling and data collection

Ear samples from five Large White pigs were used to clone the full-length *MSRB3* cDNA. For tissue distribution studies of porcine *MSRB3* mRNA transcripts, samples were collected from the heart, liver, lung, kidney, spleen, ear, muscle, fat, lymph, bone, and hypothalamus of one Large White. Ear samples from 60-day old Large White pigs (*n* = 5) and 60-day old Minzhu individuals (*n* = 5) were used to analyze the mRNA differential expression of *MSRB3* between genotypes. All samples were collected and immediately dipped into liquid nitrogen. Large White founders (*n* = 4) with small ears and Minzhu founders (*n* = 4) with large ears from a Large White × Minzhu population (reared at the pig farm of the Institute of Animal Science at the Chinese Academy of Agricultural Sciences) were used for SNP discovery. In addition, association studies were performed using 370 F2 individuals at 240 days of age from the Large White × Minzhu population and 380 Beijing Black pigs at 100 kg body weight (reared at the pig farm of Beijing Hei6 Co., Ltd, China). Left ear outline of each F2 progeny was delineated on a tracing paper using pencil and scanned by Lenovo (legend) M700 multi-function printer. Ear areas were calculated separately by Adobe Photoshop CS5.

### Genomic DNA and total RNA isolation

Genomic DNA (gDNA) was extracted from ear tissue samples as described in [9] and diluted to 50 ng/μL. The gDNA samples were stored at -20 °C and/or at 4 °C. Total RNA was isolated from tissue samples using an RNAprep Pure Tissue Kit (TIANGEN, China) according to manufacturer’s instructions. Extracted RNA was eluted in water and quantified spectrophotometrically with A260/280 ratio using NanoDrop UV-Vis (Thermo Scientific).

### Molecular cloning of full-length *MSRB3* cDNA

Reverse transcription was performed on *MSRB3* mRNA using a PrimeScript^TM^ RT reagent kit (TakaRa, Japan) according to manufacturer’s instructions. Primers were designed using the predicted porcine *MSRB3* mRNA sequence (GenBank accession no. XM_005663956) as the reference (Table 1). The part cDNA fragment was obtained using polymerase chain reaction (PCR) and sequencing of pooled cDNA from five Large White pigs.

**Table 1:** Primers for PCR amplifications of porcine *MSRB3*

Primer Purpose	Primer	Primer sequence (5’-3’)	Product size ,bp	Annealing T,°C
Part fragment cloning	M1F	ACCAGCCACTCAACTACTGC	1152	61
	M1R	ACTTCCACCAGCAGAGCTTC		
	M2F	TGGTTCTAATTGCCTCAAAGG	774	59
	M2R	GCAATGTCAGGACACCCTC		
	M3F	AGCATCCAGACTATCCCAGAAG	619	59
	M3R	TACACCTCGCCATATTGACTCA		
3’-RACE	3 F1	GGGGTGGACAAGGAAGATAA	-	55
	3 F2	AAAAGCTCCTCTTCTAATGC	-	55
5’-RACE	5R1	CTTTGAGGCAATTAGAACCAG	-	60
	5R2	TACAGTTGCCTTACATTCC	-	60
	5R3	CATAAAACTGCCCTCCTACT	-	-
	5R4	CACAGGCTCGCAGGGCTACT	-	-
qRT-PCR	M4F (target)	CCTCAGGGTCATGTAGGGATAAA	155	60
	M4R (target)	TCCAGGATCTTTGTGATGTGTATATTC		60
	G1F (control)	AGGGCATCCTGGGCTACACT	166	60
	G1R (control)	TCCACCACCCTGTTGCTGTAG		60
Polymorphism discovery for exons	M345F (exon 1)	CCGTGCCCAGGAATT	345	54
	M345R (exon1)	CGGCTCAGGAAAGAGG		
	M278F (exon 2)	GTGTTCAGTTCCAGATAAAACC	278	53
	M278R (exon 2)	ACCCCAGACCAGTGCC		
	M314F (exon 3)	GGGACTGGTCTGGTCATT	314	56
	M314R (exon 3)	CGGATTCAGCGGTTGG		
	M308F (exon 4)	GCCCTTGAAAGATTTATTGG	308	52
	M308R (exon 4)	CCGGGGAGGAACAGAA		
	M190F (exon 5)	TTCCCTTTGCAGGTCAG	190	59
	M190R (exon 5)	CATAGCCCTGCTTTGAGA		
	M272F (exon 6)	GTATCTCCTCTGTTTTGGTTC	272	59
	M272R (exon 6)	TCATTGTGCTTGTCTGTCC		
	M618F (exon 7)	CGTGGTTTCCATCGTTC	618	52
	M618R (exon 7)	CAAGCACCTTCTGCCTC		
	M2F (exon 7)	TGGTTCTAATTGCCTCAAAGG	774	59
	M2R (exon 7)	GCAATGTCAGGACACCCTC		
	M3F (exon 7)	AGCATCCAGACTATCCCAGAAG	619	59
	M3R (exon 7)	TACACCTCGCCATATTGACTCA		
	M839F (exon 7)	GCTCCTCTTCTAATGCTTACT	839	54
	M839R (exon 7)	GCAGCCTATGGCAAACT		
Polymorphism discovery for promoter	M175F	GGCTTTGGAATGAGGTTT	175	53
	M175R	TTAGACGCTGTGCTAGTTGT		
	M692F	AAACAACTAGCACAGCGTC	692	50
	M692R	GCCCAAATGCCAAAA		
	M694F	TTTCAGATACCCAGCATTG	694	50
	M694R	GCCTGAAGGGGAGTGTTA		
	M754F	CCCATTACTGTGAGGAAAA	754	53
	M754R	AAAGCAGAGCCGAGCA		
	M423F	TGGCTCGCTGTCGGA	423	58
	M423R	GGCGGGCTCATGGAA		

Rapid amplification of 5’- and 3’- cDNA ends (RACE) was performed to attain *MSRB3* cDNA using a SMART^TM^ Kit (Clontech, USA) and 3’-Full RACE Core Set with PrimeScript^TM^ RTase (TaKaRa, Japan) according to manufacturer’s instructions. Primers were designed for part cDNA fragment (Table 1). For the 5’-RACE, an initial round of PCR was performed with a gene-specific primer, 5R1, followed by a second nested PCR with another gene-specific primer, 5R2. For the 3’-RACE, an initial round of PCR was performed with a gene-specific primer, 3 F1, followed by a second nested PCR with another gene-specific primer, 3 F2. RACE PCR products were analyzed on 1.5 % agarose gels stained with GoldView and purified using a TaKaRa MiniBEST Agarose Gel DNA Extraction Kit Ver. 3.0 (TaKaRa, Japan). The purified products were cloned into T-Vector pMDTM 20 (TaKaRa, Japan) using a TaKaRa DNA Ligation Kit Version 2.1 (TaKaRa, Japan) and confirmed in both forward and reverse directions using primer walking sequencing (SinoGenoMax Co., Ltd, China), with primer 3 F2 for 3’-RACE products and primers 5R2, 5R3, and 5R4 for 5’-RACE products.

### Polymorphism detection and association analyses

Primers were designed to detect single nucleotide polymorphisms (SNPs) in all exons and the 1,000-bp 5’ flanking region of *MSRB3* (Table 1). Polymorphisms were analyzed using the DNAstar [10].

Genotypes at SNPs c.-735C > T and c.2571 T > C were determined for 370 F2 individuals of a Large White × Minzhu intercross population and 380 Beijing Black pigs using matrix-assisted laser desorption/ionization–time-off light mass spectrometry (MALDI-TOF, Sequenom, USA). Primers and probes used for MALDI-TOF were listed in Table 2. Genotype and allele frequencies were calculated for the two SNPs. Genotypic effects were analyzed by least-square analysis using the GLM procedure of SAS version 9.2, according to the following animal model:

**Table 2:** Primers and probes used for genotyping

Polymorphism	Primer/probe	Sequence
c.-735C > T	M105F	5’ACGTTGGATGTATATGGAGTTGAGGCACGC3’
	M105R	5’ACGTTGGATGGGTGAAAAGAACGACTGACC3’
	Probe105	5’CTGACCTAGATAAAACATCAG3’
c.2571 T > C	M113F	5’ACGTTGGATGAGCCTGAGGTGAAACATCTG3’
	M113R	5’ACGTTGGATGACTCGTCATTGTCACATGGG3’
	Probe113	5’ACATTGTGCTCTTCCTCT3’

$$ \mathrm{Y} = \upmu + \mathrm{G} + \mathrm{S} + \mathrm{B} + \mathrm{W} + \mathrm{e} $$

Where Y is the observation of ear size; μ is the population mean; G is the random effect of genotype; S is the fixed effect of sex; B is the fixed effect of the slaughter batch; W is the covariate effect of weight; and e is the random residue.

### Expression analysis using quantitative real-time PCR

Tissue-specific expression analysis of porcine *MSRB3* mRNA was performed by quantitative real-time PCR (qRT-PCR) using the 7900HT Fast Real-Time PCR System (Applied Biosystems). Glyceraldehyde-3-phosphate dehydrogenase (GAPDH) was used as an endogenous control to normalize the target gene expression in 11 different tissues including heart, liver, lung, kidney, spleen, ear, muscle, fat, lymph, skeletal, and hypothalamus. Total RNA was extracted and reverse-transcribed as described above. Oligonucleotide primer pair M4F/M4R and G1F/G1R (Table 1) were designed in Primer Express version 3.0 (Applied Biosystems) and used to amplify *MSRB3* and *GAPDH*, respectively. PCR amplifications were carried out in a 20-μL volume which consist of 10 μL of 2× Power SYBRGreen Master Mix (Applied BioSystems), 0.7 μL of each primer (10 μmol/L), 1.3 μL of cDNA and 7.3 μL of ddH_2_O. Cycling conditions were an initial denaturation at 95 °C for 10 min, followed by 40 annealing cycles at 95 °C for 15 s, and a final extension at 60 °C for 60 s. The differential expression analysis of *MSRB3* mRNA between Minzhu and Large White was performed by qRT-PCR as described above.

### Sequence and bioinformatics analysis

A search for open reading frames (ORFs) and translation of the nucleotide sequences into amino acid sequences was performed using Open Reading Frame Finder (ORF Finder) in NCBI (http://www.ncbi.nlm.nih.gov/gorf/gorf.html). NCBI BLAST program (http://www.ncbi.nlm.nih.gov/BLAST/) was used to compare nucleotide sequence identities of porcine *MSRB3* cDNA sequence with those of human (NM_001193461), orangutan (NM_001257851), mouse (NM_ 177092), chicken (NM_001199578) and zebrafish (NM_001002094), and putative amino acid sequence identities with those of human (NP_001026849), orangutan (NP_001244780), mouse (NP_796066), chicken (NP_001186507), and zebrafish (NP_001002094), respectively [11, 12]. The porcine *MSRB3* exons and introns were annotated using the Ensemble Blast search program (http://asia.ensembl.org/Multi/blastview). Basic characteristics of putative MSRB3 protein were predicted using the ExPASy Proteomics Server (http://web.expasy.org/), CBS Prediction Servers (http://www.cbs.dtu.dk/services/), PSORT II (http://psort.hgc.jp/form2.html), and TMpred program (http://www.ch.embnet.org/software/TMPRED_form.html).

The molecular weights and isoelectric points of multiple phosphorylation states were calculated with Scansite (http://scansite.mit.edu/calc_mw_pi.html). Secondary structure and domain were predicted by SOPMA (https://npsa-prabi.ibcp.fr/cgi-bin/npsa_automat.pl?page=/NPSA/npsa_sopma.html) and SMART (http://smart.embl-heidelberg.de/). The putative amino acid sequence of porcine MSRB3 was aligned with the species mentioned above using CLUSTAL-X program [13]. A neighbor-joining phylogenetic tree was constructed using MEGA 6 software.

## Results

### Cloning and sequencing of porcine *MSRB3* cDNA

**Fig. 1:**
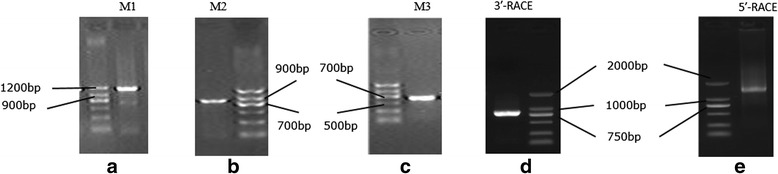
Gel electrophoresis results of PCR products for full-length analysis of porcine *MSRB3* cDNA. **a**, **b** and **c** are gel electrophoresis results for part cDNA sequences by using primers M1, M2 and M3, respectively. **d** and **e** are gel electrophoresis results of 3’-RACE and 5’-RACE. Markers in A, B and C are MarkerII(TIANGEN, China). Marks in D and E are DL2000TM DNA Marker (TaKaRa, Japan)

**Fig. 2:**
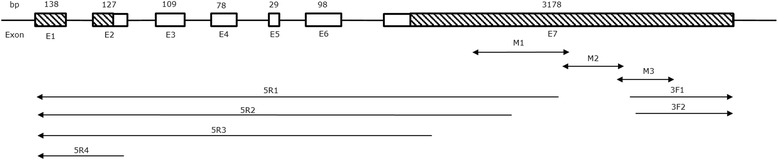
Schematic representation of porcine *MSRB3* mRNA. Exons are shown as boxes. Shaded areas represent 5’-UTR and 3’-UTR. Numbers below the boxes indicate the exon number. Numbers above the boxes are exon lengths (in bp)

Three partial cDNA fragments was amplified and identified based on the predicted porcine *MSRB3* mRNA sequence (Fig. 1a-c). A 2,266-bp internal cDNA fragment was obtained by assembling the three cDNA fragments using DNAMAN software. A 778 bp 3’-end and a 1,378 bp 5’-end were obtained by 3’-RACE and 5’-RACE, respectively (Fig. 1d and e). A 3,765 bp *MSRB3* cDNA sequence was assembled using DNAMAN software and submitted to GenBank (accession no. KP772260). Intron-exon boundaries for the porcine *MSRB3* gene were determined using Ensemble Blast, and seven exons, ranging in size from 29 to 3,178 bp, were identified (Table 3). Porcine *MSRB3* contained a 5’-UTR (190 bp), a coding region (552 bp) that encoded a 183-amino acid protein, and a 3’-UTR (3,016 bp) (Fig. 2). Additionally, the 3’-UTR contained a normal AATAAA polyadenylation signal sequence. Comparative sequence analysis revealed that identities of porcine *MSRB3* cDNA with human, orangutan, mouse, chicken and zebrafish were 84 %, 84 %, 87 %, 86 % and 70 %, respectively.

**Table 3:** Exon-intron boundaries of the porcine *MSRB3* gene

Exon/intron	Exon size ,bp	5’ Splice donor	3’ Splice acceptor
1	138	GCTCAGTCGGgtgagttggg	tcctactcagCTCTTGCCCC
2	127	CTTCCCTCAGgttgctgctt	ctctttccagGGTCATGTAG
3	109	GGACCGAAAGgtaaggcgag	tatgtttcagTGCCTTCGAA
4	78	CGTTGTTCAAgtaagtatgt	ccctttgcagGTCAGAAACA
5	29	TCTGGTTCAGgtatgtttacatta	tgtaacccagGTTGGCCTT
6	101	CTGCTCTCAGgtcagttaac	tctcttgcagTGCGGTGCTCAC
7	3,178	TTTATAAAAA	

Exon sequences are shown in uppercase letters, and intron sequences are shown in lowercase letters

Conserved GT–AG junctions are marked in boldface type

Splice junction positions are determined from our own porcine *MSRB3* sequence and the publicly available *MSRB3* genomic sequence

### Polymorphism detection and association analysis

Three SNPs were detected, one (c.2571 T > C) in the 3’-UTR, one (c.484 T > C) in the coding region and one (c.-735C > T) in the 5’ flanking region. Considering that it is a synonymous mutation, c.484 T > C was not used for subsequent analysis. A total of 370 F2 individuals and 380 Beijing Black pigs were used to perform genotyping at the other two SNPs (c.-735C > T and c.2571 T > C). Genotypic frequencies and allelic frequencies were listed in Table 4. Associations of SNP genotypes with ear size were presented in Table 4. In the F2 population, these two polymorphisms were significantly associated with ear size (*P* < 0.01). For the c.-735C > T SNP, homozygous TT individuals had least square mean (LSMean) of ear areas 22.580 and 58.261 cm^2^ larger than the heterozygous CT and homozygous CC individuals (*P* < 0.01). With regard to the c.2571 T > C SNP, LSMean ear areas of the individuals, which are CC genotype, were 39.759 and 74.738 cm^2^ larger than those with TC and TT genotype (*P* < 0.01). In Beijing Black pigs, SNPs c.-735C > T and c.2571 T > C were all not associated with ear size (*P* > 0.05).

**Table 4:** Genotype frequency and association of genotypes at the two polymorphisms with ear sizes in 370 F2 individuals and 380 Beijing Black pigs

Population	Loci	Genotype	Genotype Frequency	Mean	LSMean^1^
F2 individuals	c.-735C > T	CC	0.781	234.203	237.887 ± 49.589^a^
		CT	0.080	255.560	273.568 ± 51.129^b^
		TT	0.139	289.130	296.148 ± 48.980^c^
	c.2571 T > C	TT	0.240	209.875	211.118 ± 43.632^a^
		TC	0.490	243.947	246.097 ± 48.035^b^
		CC	0.270	277.148	285.856 ± 49.772^c^
Beijing Black pigs	c.-735C > T	CC	0.831	133.963	134.141 ± 15.999
		CT	0.076	137.201	137.466 ± 11.131
		TT	0.093	134.778	135.433 ± 14.309
	c.2571 T > C	TT	0.183	134.329	134.313 ± 15.345
		TC	0.452	134.745	135.395 ± 16.920
		CC	0.365	133.691	133.537 ± 13.809

^1^the different letters (within same common) in lower case superscript show statistically significant differences the genotype classes (*P* < 0.01)

### The mRNA expression of *MSRB3* using qRT-PCR

**Fig. 3:**
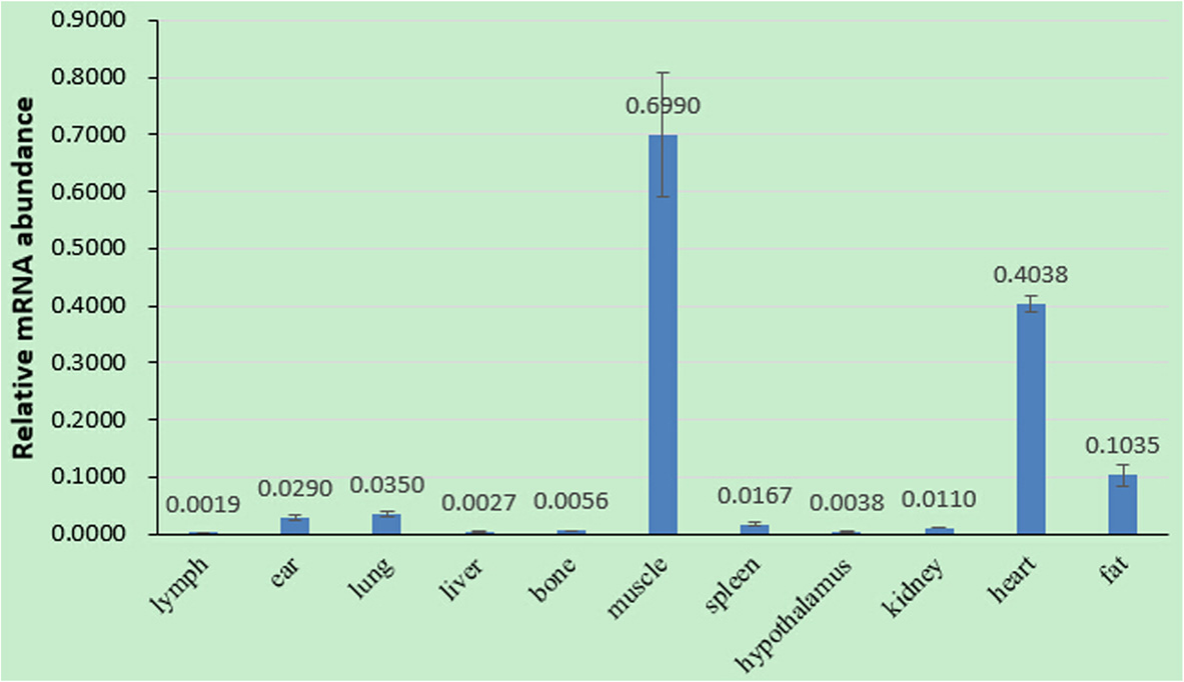
Relative mRNA transcript abundance of the porcine *MSRB3* in 11 tissues

**Fig. 4:**
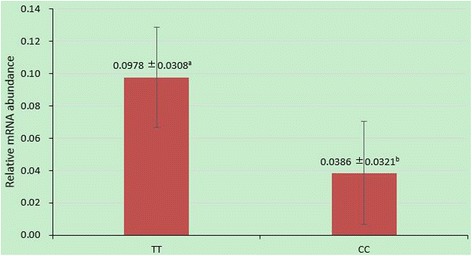
The differential expression analysis of mRNA of *MSRB3* in ear tissues. TT: Minzhu individuals with the TT genotype at SNP c.-735C > T. CC: Large White pigs with the CC genotype at SNP c.-735C > T. The different letters (within same common) in lower case superscript show statistically significant differences the genotype classes (*P* < 0.05)

The qRT-PCR analysis was conducted to further identify the tissue mRNA expression pattern of porcine *MSRB3*. The relative mRNA expression levels of *MSRB3* in 11 tissues from a subject were normalized with housekeeping gene *GAPDH*. We found that *MSRB3* mRNA was detected to be expressed among all of 11 tissues (Fig. 3). Subsequently, according to the genotype of c.-735C > T, we monitored the relative mRNA expression of *MSRB3* in ear tissues of Minzhu (TT) and Large White (CC) pigs using qRT-PCR (Fig. 4). The expression levels were significant higher in Minzhu individuals compared with Large White individuals (*P* < 0.05).

### Bioinformatic analysis of the deduced amino acid sequence

The amino acid composition of porcine predicted MSRB3 was shown in Additional file 1: Table S1 and shared 88 %, 91 %, 89 %, 86 % and 67 % identities with human, orangutan, mouse, chicken, and zebrafish respectively. Primary structure analysis revealed that the molecular weight of the putative MSRB3 protein with a theoretical isoelectric point of 7.06 was 19.8 kDa and the instability index was 48.67, which classified it as unstable by ProtParam. Amino acid content of the protein was highest in Serine (Ser) and lowest for tryptophan (Trp). Hydrophobicity analysis indicated that MSRB3 protein was strongly hydrophilic (Additional file 2: Figure S1). Seven putative O-glycosylation sites were identified at positions 13, 25, 27, 164, 165, 166 and 170 of porcine MSRB3 (Additional file 3: Figure S2) using NetOGlyc 4.0 server. According to NetPhos 2.0 output, 10 serines (Ser^27,39,62,89,95,101,118,164,166,170^), 5 threonines (Thr^48,69,126,146,178^) and 3 tyrosines (Tyr^68,77,119^) were identified as potential phosphorylation sites (Additional file 4: Figure S3). No cleavage site, N-glycosylation site or transmembrane helices were predicted. Prediction by PSORT II suggested that this protein was most likely localized in the nucleus. The molecular weights and isoelectric points of multiple phosphorylation states ranged from 19.8 to 21.2 kDa and 4.25 to 7.06, separately (Additional file 5: Table S2). Alpha helix, extended strands and random coils accounted for 17.49 %, 26.78 % and 55.74 % of the predicted secondary structure, respectively (Additional file 6: Figure S4). A SelR domain from 40^th^ to 162^th^ amino acids was predicted by SMART (Additional file 7: Figure S5).

Based on the alignment of six kinds of MSRB3 variants from human, orangutan,mouse, chicken, zebrafish and pig published in GenBank, a neighbor-joining phylogenetic tree was constructed in MEGA 6 (Additional file 8: Figure S6). The porcine MSRB3 showed a closer genetic relationship with those of human, orangutan and mouse than of the other species.

## Discussion

In this study, the complete mRNA sequence of porcine *MSRB3* was obtained. Splice donor and acceptor consensus sequences were identified at intron-exon boundaries according to the GT-AG rule [14, 15]. Human *MSRB3* can encode two protein isoforms, MSRB3A and MSRB3B, as alternative first exon splicing introduces contrasting N-terminal signal peptides [16]. In mouse, however, no alternative splicing mechanisms have been found [17]. Similar to mouse, our RACE results also indicate a lack of alternative splicing in porcine *MSRB3*. In contrast to human *MSRB3*, which contains 6–8 exons [16], pig and mouse *MSRB3* contains 7 exons [17]. Porcine MSRB3 protein shared higher amino acid sequence identities (88, 91 and 89 %) with those of human, orangutan and mouse than with chicken and zebrafish. These results suggested that MSRB3 is highly evolutionarily conserved in mammals and our neighbor-joining phylogeny built in MEGA corroborated this result. In this study, MSRB3 was predicted to be most likely localized in the nucleus, which is not definitive for the limited probability (43.5 %) based on the prediction. Human and mouse MSRB1 proteins are also localized to the cell nucleus [16]. However, mouse MSRB3 is an endoplasmic reticulum (ER) resident protein [17] while human MSRB3 is targeted to the ER and mitochondria [16]. Studies have demonstrated that zinc is indispensable for *Drosophila* MSRB protein catalysis and structure [18, 19]. Four conserved cysteine residues organized in two CxxC motifs are putative zinc-binding residues in the predicted SelR domain, suggesting that this protein is a putative zinc-containing enzyme. The *MSRB3* genes of human and mouse have been reported to be expressed ubiquitously in many tissues, including the brain, ear, lung, heart, kidney, liver, muscle, and spleen [20, 21]. Similar to our findings in *Sus scrofa*, *MSRB3* is expressed at elevated levels in heart and skeletal muscle in human and mouse [19, 22]. *MSRB3* plays an important role in regulation of cell cycle progression and cell proliferation [23]. We know that the outer ear primarily consists of skin and cartilage. Therefore, *MSRB3* may influence the skin and cartilage cell growth of the outer ear and play an important role in porcine ear size.

A SNP (H3GA0016181), which is 152199-bp away from c.2571 T > C and 319883-bp away from c.-735C > T, is significantly associated with porcine ear size in a previous study [6], and was also identified by other studies [24–26]. In humans, functional null mutations c.265 T > G and c.55C > T in *MSRB3* are associated with deafness DFNB74 [20]. In dogs, the most strongly associated SNP with ear morphology is also near *MSRB3* [7, 8]. Therefore, *MSRB3* should be regarded as a good candidate on SSC5 for porcine ear size. In the genome of *Sus scrofa*, the *MSRB* family includes three genes, designated *MSRB1*, *MSRB2*, and *MSRB3,* the latter of which is a pleiotropic gene. Besides ear, *MSRB3* plays an important role in heat, cold and oxidative tolerance, and also in the regulation of aging in transgenic drosophila lines [27]. Evidence indicates that *MSRB3* influences hippocampal size in humans [28]. Human *MSRB3* carries antimicrobial activity [29] and can inhibit the growth of *E. coli* cells when overexpressed [16]. However, it is important to note that a role of the MSRB3 protein in ear size has not been confirmed in any species until now.

In this research, three SNPs were identified in the full-length cDNA and the 5’ flanking region of *MSRB3*. Of these polymorphisms, c.2571 T > C and c.-735C > T had presented in the NCBI dbSNP (NCBI Assay ID: rs326411202 and rs340841870). Although the c.-735C > T site was a predicted transcription factor binding site for GATA1 (http://diyhpl.us/~bryan/irc/protocol-online/protocol-cache/TFSEARCH.html), the sequence around this mutation didn’t exactly match the GATA1 motif [30]. However, in this work, the mRNA of *MSRB3* was significantly higher expressed in Minzhu (TT of c.-735C > T) ears than in Large White (CC of c.-735C > T) ears. A previous report indicated that the deletion of *MSRB3* leads to cell cycle arrest at the G1 phase by activating the p53–p21 and p27 pathways in mouse embryonic fibroblast cells and this inhibitory effect on cell proliferation was also observed in primary human dermal fibroblasts [23]. Similar to the above study, the individuals (TT of c.-735C > T) with higher expression of *MSRB3* have larger ear sizes than those with CC of c.-735C > T in current research. While no significant association between c.-735C > T and ear size in Beijing Black pigs suggested *MSRB3* c.-735C > T might be linked with the QTN of ear size in F2 population other than in Beijing Black pigs. Additionally, due to the limitation number of the subjects which were used to detect SNPs, the possibility of new SNPs within this gene associated with ear size cannot be fully excluded. Hence further functional assays are still required to identify the mechanism of *MSRB3* influencing ear size in pigs.

## Conclusions

This study cloned a full-length cDNA of porcine *MSRB3*. The gene included seven exons. Tissue expression analysis indicated that *MSRB3* is highly expressed in muscle and cardiac tissue and moderately expressed in ear. Further, two SNPs were identified within gene and 5’ flanking region, showing significant association with ear size in a Large White × Minzhu F2 population. In addition, the *MSRB3* gene was significantly higher expressed in individuals with the genotype TT than those with the genotype CC at SNP c.-735C > T. Our work provides a strong molecular foundation for the genetic basis underlying porcine ear size and is expected to result in novel insights into the mechanism of *MSRB3* influencing ear size in pigs.

## Additional files

Additional file 1: Table S1. Amino acids encoded by the porcine *MSRB3* gene. (XLS 23 kb) Additional file 2: Figure S1. Kyte and Doolittle hydrophobicity plot of the putative porcine MSRB3 protein. (JPEG 56 kb) Additional file 3: Figure S2. Putative O-glycosylation sites of the putative porcine MSRB3 protein. (JPEG 152 kb) Additional file 4: Figure S3. Predicted phosphorylation sites of the putative porcine MSRB3 protein. (JPEG 32 kb) Additional file 5: Table S2. The molecular weights and isoelectric points of multiple phosphorylation states. (XLS 23 kb) Additional file 6: Figure S4. Predicted secondary structures of the deduced amino acid sequences of MSRB3. (JPEG 88 kb) Additional file 7: Figure S5. Predicted SelR domain of porcine MSRB3 by SMART. (JPEG 38 kb) Additional file 8: Figure S6. A neighbor-joining phylogenetic tree of MSRB3 species constructed in MEGA 6. The bottom scale indicates 0.05 substitutions per site. (JPEG 27 kb)

## References

[1] Ruvinsky A, Rothschild MF, Rothschild MF, Ruvinsky A (1998). Systematics and Evolution of the Pig. The Genetics of the Pig.

[2] Wei WH, de Koning DJ, Penman JC, Finlayson HA, Archibald AL, Haley CS (2007). QTL modulating ear size and erectness in pigs. Anim Genet.

[3] Ma J, Qi W, Ren D, Duan Y, Qiao R, Guo Y (2009). A genome scan for quantitative trait loci affecting three ear traits in a White Duroc x Chinese Erhualian resource population. Anim Genet.

[4] Ren J, Duan Y, Qiao R, Yao F, Zhang Z, Yang B (2011). A missense mutation in PPARD causes a major QTL effect on ear size in pigs. PLoS Genet.

[5] Li P, Xiao S, Wei N, Zhang Z, Huang R, Gu Y (2012). Fine mapping of a QTL for ear size on porcine chromosome 5 and identification of high mobility group AT-hook 2 (HMGA2) as a positional candidate gene. Genet Sel Evol.

[6] Zhang L, Liang J, Luo W, Liu X, Yan H, Zhao K (2014). Genome-wide scan reveals LEMD3 and WIF1 on SSC5 as the candidates for porcine ear size. PLoS One.

[7] Vaysse A, Ratnakumar A, Derrien T, Axelsson E, Rosengren Pielberg G, Sigurdsson S (2011). Identification of genomic regions associated with phenotypic variation between dog breeds using selection mapping. PLoS Genet.

[8] Boyko AR, Quignon P, Li L, Schoenebeck JJ, Degenhardt JD, Lohmueller KE (2010). A simple genetic architecture underlies morphological variation in dogs. PLoS Biol.

[9] Mullenbach R, Lagoda PJ, Welter C (1989). An efficient salt-chloroform extraction of DNA from blood and tissues. Trends Genet.

[10] Burland TG (2000). DNASTAR’s Lasergene sequence analysis software. Methods Mol Biol.

[11] Mount DW. Using the Basic Local Alignment Search Tool (BLAST). CSH Protoc. 2007;2007:pdb.top17. doi:10.1101/pdb.top17. 10.1101/pdb.top17 21357135

[12] Altschul SF, Gish W, Miller W, Myers EW, Lipman DJ (1990). Basic local alignment search tool. J Mol Biol.

[13] Thompson JD, Gibson TJ, Plewniak F, Jeanmougin F, Higgins DG (1997). The CLUSTAL_X windows interface: flexible strategies for multiple sequence alignment aided by quality analysis tools. Nucleic Acids Res.

[14] Burset M, Seledtsov IA, Solovyev VV (2000). Analysis of canonical and non-canonical splice sites in mammalian genomes. Nucleic Acids Res.

[15] Smith CW, Valcarcel J (2000). Alternative pre-mRNA splicing: the logic of combinatorial control. Trends Biochem Sci.

[16] Kim HY, Gladyshev VN (2004). Methionine sulfoxide reduction in mammals: characterization of methionine-R-sulfoxide reductases. Mol Biol Cell.

[17] Kim HY, Gladyshev VN (2004). Characterization of mouse endoplasmic reticulum methionine-R-sulfoxide reductase. Biochem Biophys Res Commun.

[18] Kumar RA, Koc A, Cerny RL, Gladyshev VN (2002). Reaction mechanism, evolutionary analysis, and role of zinc in Drosophila methionine-R-sulfoxide reductase. J Biol Chem.

[19] Kim HY, Gladyshev VN (2007). Methionine sulfoxide reductases: selenoprotein forms and roles in antioxidant protein repair in mammals. Biochem J.

[20] Ahmed ZM, Yousaf R, Lee BC, Khan SN, Lee S, Lee K (2011). Functional null mutations of MSRB3 encoding methionine sulfoxide reductase are associated with human deafness DFNB74. Am J Hum Genet.

[21] Marchetti MA, Pizarro GO, Sagher D, Deamicis C, Brot N, Hejtmancik JF (2005). Methionine sulfoxide reductases B1, B2, and B3 are present in the human lens and confer oxidative stress resistance to lens cells. Invest Ophthalmol Vis Sci.

[22] Kwon TJ, Cho HJ, Kim UK, Lee E, Oh SK, Bok J (2014). Methionine sulfoxide reductase B3 deficiency causes hearing loss due to stereocilia degeneration and apoptotic cell death in cochlear hair cells. Hum Mol Genet.

[23] Lee E, Kwak GH, Kamble K, Kim HY (2014). Methionine sulfoxide reductase B3 deficiency inhibits cell growth through the activation of p53-p21 and p27 pathways. Arch Biochem Biophys.

[24] Li P (2012). Fine mapping of the QTL for ear size on pig chromosome 5 and preliminary identification of the causative gene.

[25] Liu C (2013). Preliminary identification of the causative gene and mutation for QTL influencing ear size on pig chromosome 5.

[26] Qiao R (2014). Understanding the molecular mechanism of external ear innate defect by using pig as a model.

[27] Lim DH, Han JY, Kim JR, Lee YS, Kim HY (2012). Methionine sulfoxide reductase B in the endoplasmic reticulum is critical for stress resistance and aging in Drosophila. Biochem Biophys Res Commun.

[28] Bis JC, DeCarli C, Smith AV, van der Lijn F, Crivello F, Fornage M (2012). Common variants at 12q14 and 12q24 are associated with hippocampal volume. Nat Genet.

[29] Kim Y, Kwak GH, Lee C, Kim HY (2011). Identification of an antimicrobial peptide from human methionine sulfoxide reductase B3. BMB Rep.

[30] Evans T, Reitman M, Felsenfeld G (1988). An erythrocyte-specific DNA-binding factor recognizes a regulatory sequence common to all chicken globin genes. Proc Natl Acad Sci U S A.

