# Supplementary figures and images for: Porcine methionine sulfoxide reductase B3: molecular cloning, tissue-specific expression profiles, and polymorphisms associated with ear size in Sus scrofa
Source: J Anim Sci Biotechnol. 2015 Dec 30;6:60. doi: 10.1186/s40104-015-0060-x (PMC4696113; doi:10.1186/s40104-015-0060-x)

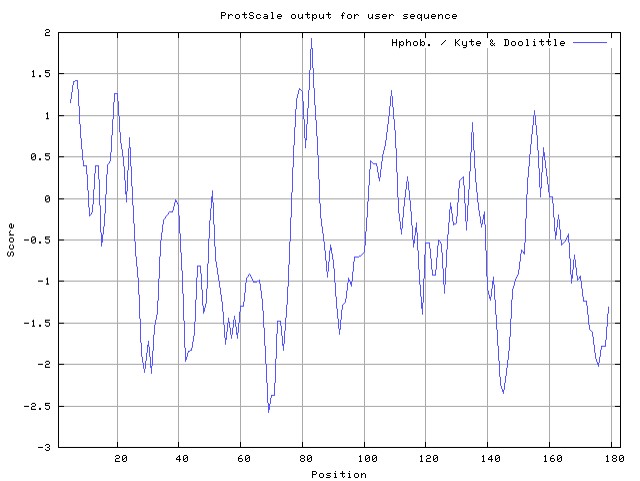

Supplement: Additional file 2: Figure S1. — Kyte and Doolittle hydrophobicity plot of the putative porcine MSRB3 protein. (JPEG 56 kb) [file 40104_2015_60_MOESM2_ESM.jpeg]

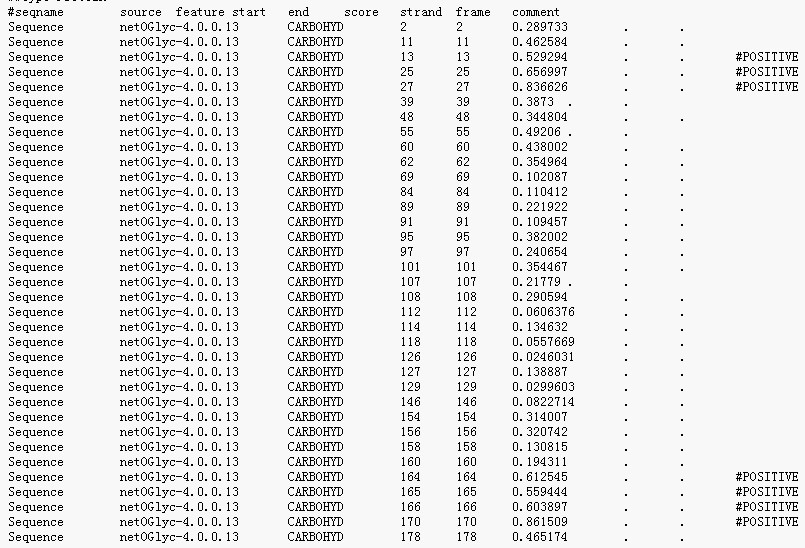

Supplement: Additional file 3: Figure S2. — Putative O-glycosylation sites of the putative porcine MSRB3 protein. (JPEG 152 kb) [file 40104_2015_60_MOESM3_ESM.jpeg]

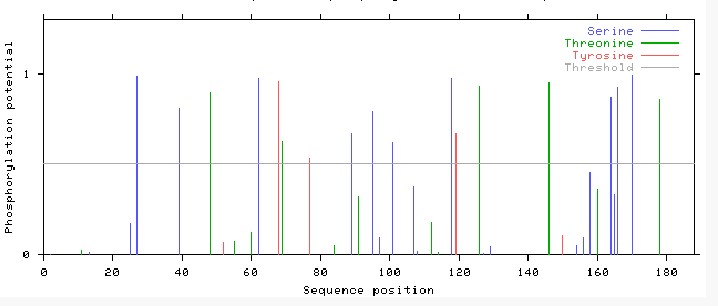

Supplement: Additional file 4: Figure S3. — Predicted phosphorylation sites of the putative porcine MSRB3 protein. (JPEG 32 kb) [file 40104_2015_60_MOESM4_ESM.jpeg]

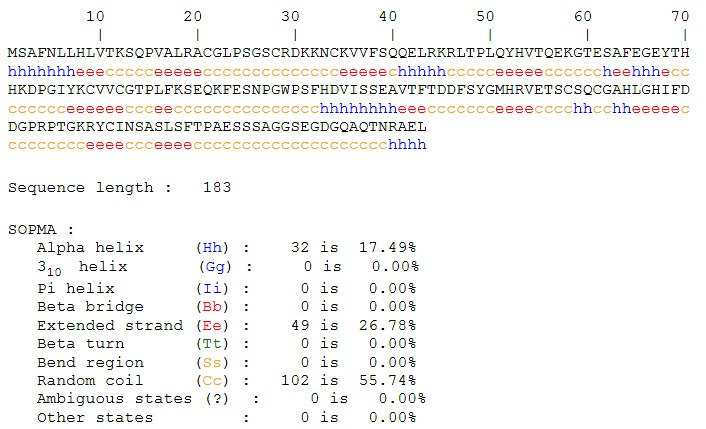

Supplement: Additional file 6: Figure S4. — Predicted secondary structures of the deduced amino acid sequences of MSRB3. (JPEG 88 kb) [file 40104_2015_60_MOESM6_ESM.jpeg]

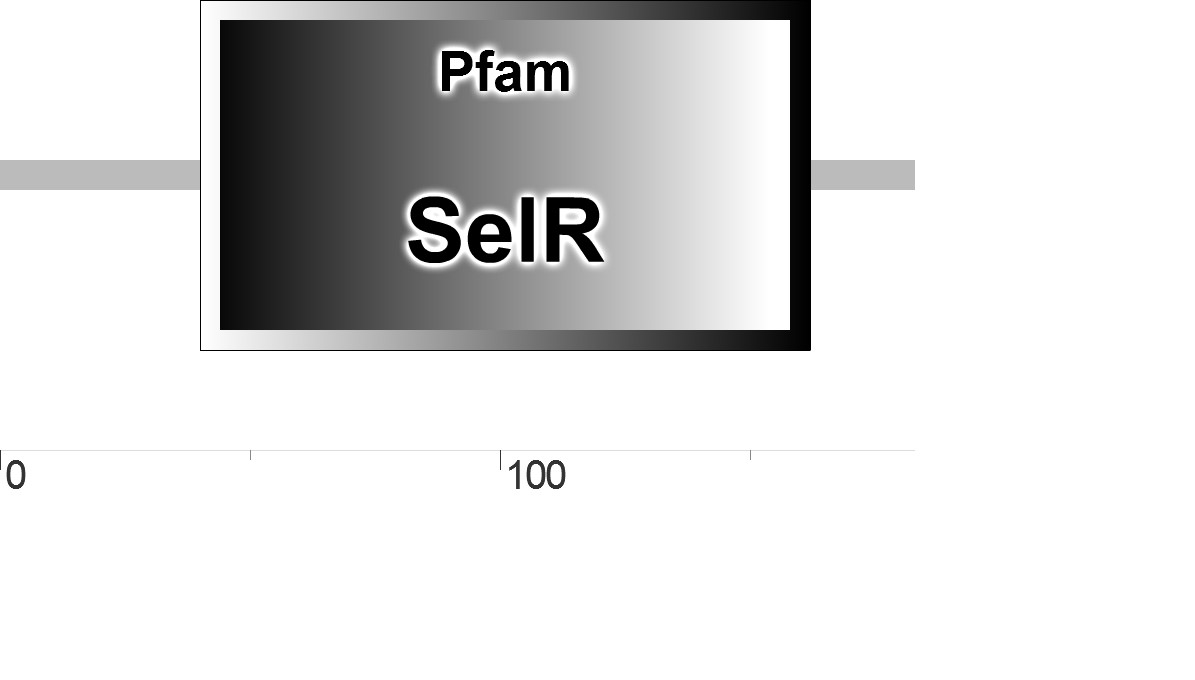

Supplement: Additional file 7: Figure S5. — Predicted SelR domain of porcine MSRB3 by SMART. (JPEG 38 kb) [file 40104_2015_60_MOESM7_ESM.jpeg]

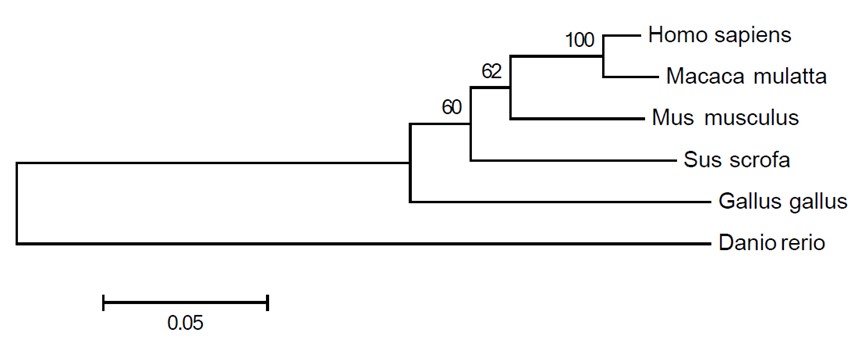

Supplement: Additional file 8: Figure S6. — A neighbor-joining phylogenetic tree of MSRB3 species constructed in MEGA 6. The bottom scale indicates 0.05 substitutions per site. (JPEG 27 kb) [file 40104_2015_60_MOESM8_ESM.jpeg]
